# Jahresbericht 2025 aus der Kerndokumentation der Regionalen Kooperativen Rheumazentren

**DOI:** 10.1007/s00393-025-01646-8

**Published:** 2025-05-05

**Authors:** Katinka Albrecht, Katja Thiele, Tobias Alexander, Martin Aringer, Jacqueline Detert, Thorsten Eidner, Martin Feuchtenberger, Jörg Henes, Kirsten Karberg, Uta Kiltz, Benjamin Köhler, Andreas Krause, Jutta G. Richter, Susanna Späthling-Mestekemper, Mirko Steinmüller, Silke Zinke, Anja Strangfeld, Johanna Callhoff

**Affiliations:** 1 Programmbereich Epidemiologie und Versorgungsforschung, Deutsches Rheuma Forschungszentrum Berlin, Charitéplatz1, 10117 Berlin, Deutschland; 2 Medizinische Klinik m.S. Rheumatologie und Klinische Immunologie, Charité – Universitätsmedizin Berlin, corporate member Freie Universität Berlin, Humboldt-Universität zu Berlin, und Berlin Institute of Health (BIH), Berlin, Deutschland; 3 Autoimmunology Group, Deutsches Rheuma-Forschungszentrum Berlin, Berlin, Deutschland; 4 Bereich Rheumatologie, Medizinische Klinik und Poliklinik III und UniversitätsCentrum für Autoimmun- und Rheumatische Erkrankungen (UCARE) Universitätsklinikum und Medizinische Fakultät Carl Gustav Carus, Technische Universität Dresden, Dresden, Deutschland; 5 Rheumapraxis Templin, Templin, Templin, Deutschland; 6 Klinik für Innere Medizin III – Rheumatologie/Osteologie, Universitätsklinikum Jena, Jena, Deutschland; 7 Rheumatologie, MVZ MED BAYERN OST, Burghausen, Deutschland; 8 Medizinische Klinik und Poliklinik II, Universitätsklinikum Würzburg, Würzburg, Deutschland; 9 Zentrum für Interdisziplinäre Rheumatologie, klinische Immunologie und Autoimmunerkrankungen (INDIRA), Department für Innere Medizin II, Universitätsklinik Tübingen, Tübingen, Deutschland; 10 Rheumatologisches Versorgungszentrum Steglitz, Berlin, Deutschland; 11 Ruhr-Universität Bochum, Bochum, Deutschland; 12 Rheumazentrum Ruhrgebiet, Herne, Deutschland; 13 Rheumazentrum Ratingen, Rheumatologische Gemeinschaftspraxis, Ratingen, Deutschland; 14 Rheumatologie, klinische Immunologie und Osteologie, Immanuel-Krankenhaus Berlin, Berlin, Deutschland; 15 Klinik für Rheumatologie, Medizinische Fakultät, Universitätsklinikum Düsseldorf, Heinrich-Heine-Universität Düsseldorf, Düsseldorf, Deutschland; 16 Hiller Forschungszentrum Rheumatologie, Medizinische Fakultät, Universitätsklinikum Düsseldorf, Heinrich-Heine-Universität Düsseldorf, Düsseldorf, Deutschland; 17 Rheumapraxis München-Pasing, München, Deutschland; 18 Rheumateam Lahn-Dill-Siegerland – Wetzlar/Burbach, Wetzlar/Burbach, Deutschland; 19 Rheumatologische Schwerpunktpraxis Berlin, Berlin, Deutschland; 20 Medizinische Klinik mit Schwerpunkt Rheumatologie und Klinische Immunologie, Charité-Universitätsmedizin Berlin, Berlin, Deutschland

**Keywords:** Versorgungsforschung, Rheumatologie, Rheumatische Erkrankungen, Langzeitbeobachtung, „Disease-modifying antirheumatic drugs“(DMARD)-Therapie, Health services research, Rheumatology, Rheumatic diseases, Long-term observational research, DMARD therapy

## Abstract

**Hintergrund:**

Die Kerndokumentation der Regionalen Kooperativen Rheumazentren erfasst jährlich Versorgungsdaten von Patient:innen mit entzündlich rheumatischen Erkrankungen.

**Methodik:**

Für rheumatoide Arthritis (RA), Psoriasisarthritis (PsA), axiale Spondyloarthritis (axSpA), systemischen Lupus erythematodes (SLE), systemische Sklerose (SSc), Sjögren-Syndrom (SjS), idiopathische inflammatorische Myositiden (IIM), Polymyalgia rheumatica (PMR), Riesenzellerarteriitis (RZA), ANCA-assoziierte Vaskulitiden (AAV), Morbus Behçet (BD), „adult-onset Still’s disease“ (AOSD) und autoinflammatorische Erkrankungen (AIE) werden Daten aus dem Jahr 2023 berichtet. Angaben umfassen u. a. die ärztlich eingeschätzte Krankheitsaktivität auf einer numerischen Ratingskala (NRS) von 0–10, Therapien und patientenberichtete Outcomes. Für ausgewählte Diagnosen werden Entwicklungen von 2010 bis 2023 zu der ärztlichen Einschätzung der Krankheitsaktivität und zu Therapien dargestellt.

**Ergebnisse:**

Aus 14 Einrichtungen wurden 13.884 Patient:innen dokumentiert, am häufigsten RA (5734), PsA (1741) und axSpA (1494). Das mittlere Alter lag zwischen 45 (BD) und 73 Jahren (RZA), die Krankheitsdauer betrug im Median 3 (PMR) bis 16 Jahre (axSpA). Die Krankheitsaktivität war überwiegend niedrig, bei 6 % (BD) bis 15 % (axSpA) stuften die Rheumatolog:innen sie moderat bis hoch (> 4 auf numerischer Ratingskala) ein. Biologische (b)DMARDs wurden am häufigsten bei axSpA (65 %), AOSD (58 %), PsA (53 %) und RZA (41 %) verordnet. Häufig waren Tumornekrosefaktor(TNF)-Inhibitoren bei axSpA (53 %), BD (30 %) und PsA (28 %), Interleukin(IL)-1-Inhibitoren bei AOSD (51 %) und AIE (50 %), IL-6Ri bei RZA (38 %), IL-17i bei PsA (17 %) und Rituximab bei AAV (29 %). Höhere Einschränkungen hinsichtlich Schmerz, Fatigue, Schlafstörungen und Wohlbefinden dokumentierten v. a. Betroffene mit IIM, SSc, axSpA und AIE. Von den unter 65-Jährigen waren 58 % (SSc) bis 77 % (axSpA) erwerbstätig. Eine Berentung wegen der rheumatischen Erkrankung hatten 5 % (AOSD) bis 18 % (AAV). In der Entwicklung seit 2010 ist der Anteil an Patienten in Remission oder sehr niedriger Krankheitsaktivität (NRS 0–1) diagnoseübergreifend gestiegen. Bei den Therapien sind ein Anstieg an b/tsDMARDs und ein Rückgang von Glukokortikoiden bei verschiedenen Diagnosen sichtbar.

**Schlussfolgerung:**

Die Ergebnisse zeigen die Vielfältigkeit der entzündlich rheumatischen Diagnosen und das kontinuierlich wachsende Therapiespektrum in der Rheumatologie, einhergehend mit einer guten Krankheitskontrolle bei vielen Patient:innen.

## Abstract (en)

**Background:**

Data of patients with inflammatory rheumatic diseases are annually recorded within the National Database of the German Collaborative Rheumatology Centers.

**Methods:**

For rheumatoid arthritis (RA), psoriatic arthritis (PsA), axial spondylarthritis (axSpA), systemic lupus erythematosus (SLE), systemic sclerosis (SSc), Sjögren’s syndrome (SjS), idiopathic inflammatory myositis (IIM), polymyalgia rheumatica (PMR), giant cell arteritis (GCA), ANCA-associated vasculitis (AAV), Behçet’s disease (BD), adult onset Still’s disease (AOSD) and autoinflammatory diseases (AID) data are reported from 2023. Information includes physician-reported disease activity on a numeric rating scale (NRS) of 0–10, treatment and patient-reported outcomes. For selected diagnoses, developments from 2010 to 2023 are presented regarding physicians’ assessments of disease activity and treatment.

**Results:**

A total of 13,884 patients were documented from 14 rheumatology centers, most frequently with RA (5734), PsA (1741) and axSpA (1494). The mean age ranged from 45 years (BD) to 73 years (GCA) and the median disease duration ranged from 3 years (PMR) to 16 years (axSpA). Disease activity was predominantly low, with 6% (BD) to 15% (axSpA) rated moderate to high (> 4 on the NMR) by rheumatologists. Biological disease-modifying antirheumatic drugs (bDMARD) were most frequently prescribed for axSpA (65%), AOSD (58%), PsA (53%) and GCA (41%). Tumor necrosis factor (TNF) inhibitors were frequently used in axSpA (53%), BD (30%) and PsA (28%), interleukin (IL)-1 inhibitors in AOSD (51%) and AID (50%), IL-6Ri in GCA (38%), IL17i in PsA (17%) and rituximab in AAV (29%). Higher levels of pain, fatigue, sleep disturbances and reduced well-being were reported by patients with IIM, SSc, axSpA and AID. Among those younger than 65 years, 58% (SSc) to 77% (axSpA) were employed. The percentage of early retirement due to rheumatic diseases was 5% (AOSD) to 18% (AAV). Since 2010 the development in the proportion of patients in remission or with very low disease activity (NRS 0-1) has increased across all diagnoses. In terms of treatment there has been an increase in b/tsDMARDs and a decrease in glucocorticoids for various diagnoses.

**Conclusion:**

The results show the diversity of inflammatory rheumatic diagnoses and the continuously growing range of treatment in rheumatology along with good disease control in many patients.

In der Kerndokumentation der Regionalen Kooperativen Rheumazentren werden jährlich ärztlich und patientenberichtete Daten zur rheumatologischen Versorgung und Krankheitslast von Patient:innen mit entzündlich rheumatischen Erkrankungen erhoben. Der Jahresbericht beinhaltet aktuelle Daten zu Krankheitsaktivität, Therapie und patientenberichtete Outcomes für das gesamte Spektrum rheumatischer Diagnosen. Erstmals werden auch Daten für das Still-Syndrom des Erwachsenen („adult-onset Still’s disease“ [AOSD]) und für genetische autoinflammatorische Erkrankungen (AIE) berichtet. Diese umfassen u. a. familiäres Mittelmeerfieber und Cryopyrin- bzw. Tumornekrosefaktor-assoziierte periodische Fiebersyndrome. Außerdem berichten wir Daten zur beruflichen Teilhabe bei Patient:innen im erwerbsfähigen Alter. Für ausgewählte Diagnosen werden zeitliche Trends von 2010 bis 2023 zur Krankheitsaktivität und zu den Therapien dargestellt.

## Methodik

Die Kerndokumentation ist eine seit 1993 durchgeführte bundesweite prospektive Langzeitdokumentation. Patient:innen aus der rheumatologischen Routineversorgung werden 1‑mal im Jahr hinsichtlich ihres Krankheitsverlaufes dokumentiert [1]. In die Kerndokumentation können Patient:innen mit allen rheumatischen Erkrankungen eingeschlossen werden. Die Daten aus diesem Bericht beziehen sich auf das Auswertungsjahr 2023 und für die zeitlichen Trends auf die Jahre 2010 bis 2023. Für die Darstellung von Patientencharakteristika, Therapien und patientenberichteten Outcomes (PROs) sind nur Patient:innen mit einer gesicherten Diagnose berücksichtigt. Für folgende Diagnosen werden Daten ausgegeben: rheumatoide Arthritis (RA), Psoriasisarthritis (PsA), axiale Spondyloarthritis (axSpA), systemischer Lupus erythematodes (SLE), systemische Sklerose (SSc), Sjögren-Syndrom (SjS), idiopathische inflammatorische Myositiden (IIM), Polymyalgia rheumatica (PMR), Riesenzellarteriitis (RZA), ANCA-assoziierte Vaskulitiden (AAV), Morbus Behçet (BD), AOSD und AIE.

Die ärztlich eingeschätzte Krankheitsaktivität wird bei allen Krankheitsentitäten mit einer numerischen Ratingskala (NRS) von 0 bis 10 erfasst, wobei 0 keiner und 10 der höchsten Aktivität entspricht. Bei RA wird der Disease Activity Score (DAS28) bzw. der Clinical oder Simple Disease Activity Index (CDAI oder SDAI) erhoben, bei axSpA der Bath Ankylosing Spondylitis Disease Activity Index (BASDAI) und der Axial Spondyloarthritis Disease Activity Score (ASDAS). Bei SLE werden in einigen Einrichtungen der European Consensus Lupus Activity Measurement (ECLAM) und der Systemic Lupus Disease Activity Index (SLEDAI) erhoben.

Als medikamentöse Therapien werden erfasst: Glukokortikoide (GC), alle konventionell synthetischen (cs), biologischen (b) und zielgerichteten (englisch „targeted synthetic“ [ts]) immunmodulierenden Medikamente (englisch „disease-modifying antirheumatic drugs“ [DMARDs]), nichtsteroidale Antirheumatika (NSAR), nicht opioidhaltige Analgetika und Opioide. Die Abfrage nichtmedikamentöser Therapien umfasst Physiotherapie, Ergotherapie, Rheumafunktionstraining und Patientenschulung.

Patientenberichtete Outcomes (PRO) beinhalten u. a. den allgemeinen Gesundheitszustand, Krankheitsaktivität, Schmerzen, Erschöpfung/Müdigkeit, Schlafstörungen, Schwierigkeiten bei körperlichen Tätigkeiten sowie psychisches und körperliches Wohlbefinden, jeweils bezogen auf die letzte Woche. Diese werden mit einer NRS, adaptiert vom Rheumatoid Arthritis Impact of Disease (RAID) [2] erfasst. Für die Funktionsfähigkeit wird bei RA der Funktionsfragebogen Hannover (FFbH) und bei axSpA der Bath Ankylosing Spondylitis Functional Index (BASFI) erhoben.

Bezüglich der beruflichen Teilhabe dokumentieren die Patient:innen, ob sie erwerbstätig (in Voll- oder Teilzeit, arbeitslos/in Ausbildung/Umschulung) sind, ob mindestens eine Arbeitsunfähigkeit in den letzten 12 Monaten aufgrund der rheumatischen Erkrankung bestand und, falls ja, Anzahl der Tage sowie Erwerbs- oder Berufsunfähigkeit aufgrund der rheumatischen Erkrankung.

## Ergebnisse

Im Jahr 2023 erhoben 14 rheumatologische Einrichtungen (7 Praxen, 1 Krankenhaus und 6 Universitätskliniken) Daten für die Kerndokumentation. Dokumentiert wurden insgesamt 13.884 ambulant versorgte Patient:innen. Die häufigsten Krankheitsentitäten waren RA (*n* = 5734), PsA (*n* = 1741), axSpA (*n* = 1494) und SLE (*n* = 937), s. Tab. 1. Über die Hälfte der Patient:innen (62 %) wurde über die ambulante spezialfachärztliche Versorgung (ASV), 30 % über die Regelversorgung und 8 % über eine Hochschul‑/Ermächtigungsambulanz abgerechnet.

**Tab. 1:** Patientencharakteristika von 2023

	RA	PsA	axSpA	SLE	SSc	SjS	IIM	MCTD	PMR	RZA	AAV	BD	AOSD	AIE
*N*	5734	1741	1494	937	343	224	108	147	446	390	305	159	85	87
Weiblich (%)	73 %	56 %	38 %	88 %	76 %	92 %	64 %	85 %	59 %	72 %	56 %	43 %	66 %	58 %
BMI, MW (SD)	27 (6)	28 (6)	27 (5)	25 (5)	25 (5)	25 (6)	27 (6)	26 (6)	27 (5)	26 (4)	26 (6)	26 (4)	26 (6)	27 (6)
BMI ≥ 30 kg/m^2^ (%)	24 %	33 %	27 %	17 %	12 %	15 %	21 %	19 %	20 %	15 %	21 %	15 %	23 %	25 %
Alter in 2023 in Jahren, MW (SD)	62 (14)	56 (14)	50 (14)	49 (15)	58 (14)	55 (14)	56 (16)	53 (15)	71 (9)	73 (9)	61 (14)	45 (11)	48 (14)	38 (14)
Erkrankungsalter in Jahren, MW (SD)	48 (16)	42 (15)	31 (13)	31 (14)	45 (14)	42 (14)	45 (16)	37 (17)	66 (10)	67 (9)	49 (15)	28 (10)	36 (15)	17 (14)
Krankheitsdauer in 2023 in Jahren, MW (SD)	14 (11)	14 (11)	18 (13)	17 (11)	12 (9)	13 (9)	11 (8)	16 (10)	5 (7)	6 (5)	11 (8)	17 (10)	12 (10)	21 (12)
Krankheitsdauer, Median	11	11	16	16	11	12	9	16	3	4	9	16	9	20
Krankheitsdauer < 2 Jahre (%)	8 %	7 %	4 %	6 %	6 %	5 %	14 %	7 %	37 %	21 %	7 %	3 %	7 %	0 %

*RA* rheumatoide Arthritis, *PsA* Psoriasisarthritis, *axSpA* axiale Spondyloarthritis, *SLE* systemischer Lupus erythematodes, *SSc* systemische Sklerose, *SjS* primäres Sjögren-Syndrom, *IIM* idiopathische Inflammatorische Myositiden, *MCTD* „mixed connective tissue diseases“, *PMR* Polymyalgia rheumatica, *RZA* Riesenzellarteriitis, *AAV* ANCA-assoziierte Vaskulitiden, *BD* Morbus Behçet, *AOSD* „adult-onset Still’s disease“, *AIE* autoinflammatorische Erkrankungen. *MW* Mittelwert, *SD* Standardabweichung, *BMI* Body Mass Index

### Patientencharakteristika

Das mittlere Alter der Patient:innen variierte von 45 Jahren (BD) bis 73 Jahre (RZA). Die Krankheitsdauer lag im Median zwischen 3 Jahren bei PMR und 16 Jahren bei axSpA. Das mittlere Alter bei Erkrankung lag zwischen 28 Jahren bei BD und 67 Jahren bei RZA. Drei Prozent (BD) bis 37 % (PMR) hatten eine Krankheitsdauer unter 2 Jahren. Der Anteil an Frauen war mit 92 % bei primärem Sjögren-Syndrom (SjS) am höchsten und mit 38 % bei axSpA am niedrigsten.

### Krankheitsaktivität und Remission

Die auf der NRS angegebene ärztlich dokumentierte Krankheitsaktivität lag krankheitsübergreifend im niedrigen Bereich: Der Mittelwert war am niedrigsten bei BD (0,9 ± 1,3) und am höchsten bei axSpA (1,8 ± 1,8). Bei 6 (BD) bis 15 % (axSpA) stuften die Rheumatolog:innen die Aktivität als moderat bis hoch (> 4) ein, s. Tab. 2.

**Tab. 2:** Krankheitsaktivität nach ärztlicher Einschätzung

	RA	PsA	axSpA	SLE	SSc	SjS	IIM	MCTD	PMR	RZA	AAV	BD	AOSD	AIE
Krankheitsaktivität, ärztliche Einschätzung, NRS 0–10, MW (SD)	1,5 (1,7)	1,7 (1,8)	1,8 (1,8)	1,3 (1,4)	1,8 (1,5)	1,7 (1,6)	1,5 (1,6)	1,3 (1,2)	1,1 (1,4)	1,1 (1,4)	1,3 (1,5)	0,9 (1,3)	1,3 (1,8)	1,3 (1,7)
Moderat bis hoch (4–10), %	13	14	15	8	13	12	12	6	9	7	9	6	10	11

*RA* rheumatoide Arthritis, *PsA* Psoriasisarthritis, *axSpA* axiale Spondyloarthritis, *SLE* systemischer Lupus erythematodes, *SSc* systemische Sklerose, *SjS* primäres Sjögren-Syndrom, *IIM* idiopathische inflammatorische Myositiden, *MCTD* „mixed connective tissue diseases“, *PMR* Polymyalgia rheumatica, *RZA* Riesenzellarteriitis, *AAV* ANCA-assoziierte Vaskulitiden, *BD* Morbus Behçet, *AOSD* „adult-onset Still’s disease“, *AIE* autoinflammatorische Erkrankungen, *MW* Mittelwert, *SD* Standardabweichung, *NRS* numerische Ratingskala, 0 entspricht inaktiver und 10 hochaktiver Erkrankung

**Abb. 1:**
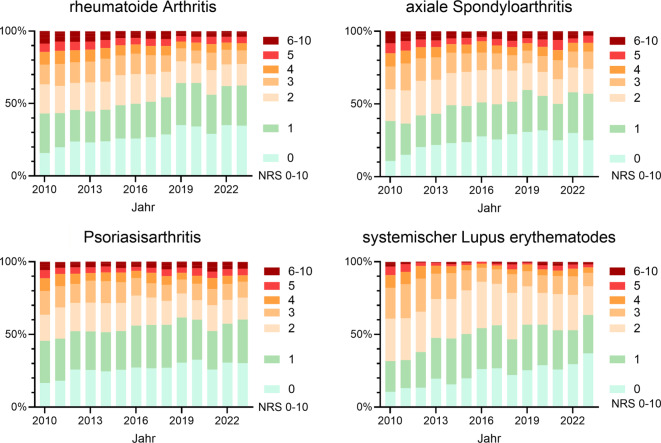
Ärztlich eingeschätzte Krankheitsaktivität auf numerischer Ratingskala von 0 (keine) bis 10 (sehr hohe Aktivität). Dargestellt ist der prozentuale Anteil an Patient:innen mit der jeweiligen Einschätzung in den Jahren 2010 bis 2023

Seit 2010 ist die Zahl der Patient:innen in Remission bzw. mit sehr niedriger Krankheitsaktivität (NRS 0 oder 1) diagnoseübergreifend gestiegen (Abb. 1).

Von den RA-Patient:innen befanden sich 47 % in DAS28-BSG-Remission (< 2,6), 19 % hatten eine niedrige (2,6–3,2), 29 % eine mittlere (> 3,2–5,1) und 5 % (> 5,1) eine hohe Krankheitsaktivität. Nach dem CDAI waren 25 % in Remission (≤ 2,8), 51 % hatten eine niedrige (2,8–10), 19 % eine moderate (11–22) und 5 % eine hohe (> 22) Krankheitsaktivität. Von den axSpA-Patient:innen hatten 22 % gemäß dem ASDAS-CRP eine inaktive Erkrankung (< 1,3), 28 % eine moderate (1,3 bis < 2,1), 43 % eine hohe (2,1–3,5) und 8 % eine sehr hohe (> 3,5) Krankheitsaktivität. Nach dem BASDAI hatten 30 % eine niedrige (0–2), 30 % eine mittlere (< 2–4) und 40 % eine hohe Krankheitsaktivität (> 4).

Der ECLAM wurde bei 96 SLE-Patientinnen erfasst. Nach dem ECLAM-Score (0–10, 0 entsprechend einer inaktiven Erkrankung) betrug der Score bei 14 % 0, bei 26 % 1, bei 50 % 2, bei 6 % 3, bei 2 % 4 und bei 2 % 5. Am häufigsten waren Fieber/Fatigue (31 %) und eine hämatologische Beteiligung (19 %).

### Glukokortikoide, NSAR und Schmerzmedikation

Im Jahr 2023 wurden 27 % der Patient:innen mit RA, 44 % mit SLE und 54 % mit AAV mit Glukokortikoiden (GC) behandelt, die Prozentzahlen für die weiteren Diagnosen sind in Tab. 3 angeführt. Davon hatten etwa 80 % eine niedrige Dosis von bis zu 5 mg Prednisolonäquivalent pro Tag. AOSD-Patient:innen hatten zu 39 % und PMR-Patient:innen zu 35 % > 5 mg pro Tag. NSAR wurden v. a. bei axSpA (64 %), PsA (43 %) und RA (41 %) eingesetzt. Sechs bis 22 % der Patient:innen erhielten nicht opioidhaltige Analgetika und 3 % (BD) bis 8 % (AOSD) nahmen ein Opioid ein.

**Tab. 3:** Konventionelle medikamentöse Therapien

	RA	PsA	axSpA	SLE	SSc	SjS	IIM	MCTD	PMR	RZA	AAV	BD	AOSD	AIE
Glukokortikoide	27	9	5	44	17	27	43	35	57	45	54	35	27	7
Davon ≤ 5 mg/Tag	79	72	74	81	71	83	78	88	65	73	84	80	61	83
NSAR	41	43	64	23	14	26	15	23	24	12	11	18	27	26
Analgetika^a^	17	14	16	17	11	17	18	19	18	22	17	13	13	6
Opioide	6,8	7,6	7,7	5,2	3,0	5,9	4,7	6,2	5,4	5,9	5,3	3,2	8,4	3,4
Basistherapie (cs/b/tsDMARD)	90	86	73	91	61	71	80	78	38	64	80	62	76	56
csDMARD	65	42	12	88	58	67	75	71	36	26	53	34	29	5
Azathioprin	0,2	0,1	0,4	17	4,3	6,3	19	10	0,5	1,6	23	23	0	1,2
Ciclosporin A	0	0,1	0	1,2	0	0,9	1,9	0	0	0	0	1,9	2,4	0
Cyclophosphamid	0	0	0	0,5	0,9	0	0	0	0	0	1,0	0	0	0
Hydroxychloroquin	5,6	0,4	0,1	76	9,0	54	6,5	50	0,7	0	0	0,6	2,4	1,2
Leflunomid	6,5	2,7	0,4	0,2	0,6	0,9	1,9	2,8	3,0	0,3	2,0	0	0	1,2
Methotrexat	53	37	7,3	9,5	25	10	42	17	32	25	27	8,9	25	2,3
Mycophenolat	0	0,1	0,1	16	21	1,8	12	4,1	0	0	2,6	0	0	0
Sulfasalazin	3,5	2,1	3,5	0,1	0	0	0	0	0,5	0	0	0,6	0	0

Alle Angaben sind Prozentwerte

*RA* rheumatoide Arthritis, *PsA* Psoriasisarthritis, *axSpA* axiale Spondyloarthritis, *SLE* systemischer Lupus erythematodes, *SSc* systemische Sklerose, *SjS* primäres Sjögren-Syndrom, *IIM* idiopathische inflammatorische Myositiden, *MCTD* „mixed connective tissue diseases“, *NSAR* nichtsteroidale Antirheumatika, *PMR* Polymyalgia rheumatica, *RZA* Riesenzellarteriitis, *AAV* ANCA-assoziierte Vaskulitiden, *BD* Morbus Behçet, *AOSD* „adult-onset Still’s disease“, *AIE* autoinflammatorische Erkrankungen

^a^Nicht opioidhaltig

### csDMARDs

Eine Therapie mit einem csDMARD, bDMARD oder tsDMARD hatten 62 % (BD) bis 91 % (SLE) der Patient:innen. Methotrexat (MTX) war das am häufigsten verwendete csDMARD (s. Tab. 3). MTX wurde v. a. bei RA (53 %), IIM (42 %) und PsA (37 %) verwendet. Azathioprin kam am häufigsten bei AAV und BD (je 23 %) zum Einsatz. Bei SLE (76 %), SjS (54 %) und MCTD (50 %) war Hydroxychloroquin (HCQ) das häufigste csDMARD. Mycophenolat-Mofetil spielte am ehesten bei SSc (21 %) und bei SLE (16 %) eine Rolle. Leflunomid (6,5 % bei RA, 2,7 % PsA), Sulfasalazin, Ciclosporin A und Cyclophosphamid waren nur bei wenigen Patient:innen Bestandteil der Therapie.

### bDMARDs

Biologische (b)DMARDs wurden am häufigsten bei axSpA (65 %), AOSD (58 %), PsA (53 %) und AIE (52 %) verordnet (s. Tab. 4). Auch bei RZA (41 %), RA (33 %), AAV (34 %) und BD (31 %) erhielt jeder dritte Patient ein bDMARD. Die am häufigsten eingesetzte bDMARD-Gruppe waren TNF-Inhibitoren (TNFi), v. a. bei axSpA (53 %), BD (30 %), PsA (28 %) und RA (21 %). Bei PsA waren auch IL-17-Inhibitoren (17 %) und IL-12/23-Inhibitoren (7 %) relevant. Patient:innen mit AOSD und AIE wurden mit den IL-1-Inhibitoren Anakinra und Canakinumab behandelt.

**Tab. 4:** Biologische DMARDs

	RA	PsA	axSpA	SLE	SSc	SjS	IIM	MCTD	PMR	RZA	AAV	BD	AOSD	AIE
bDMARD (any)	33	53	65	18	5,6	8,1	13	10	2,7	41	34	31	58	52
TNFi	21	28	53	0,4	0,6	0	0	1,4	0,9	1,1	0,7	30	3,6	2,3
– Adalimumab	8,6	15	25	0,3	0,6	0	0	0,7	0	0	0,7	7,0	2,4	1,2
– Certolizumab	2,7	2,9	4,3	0	0	0	0	0,7	0	0	0	0	0	0
– Etanercept	8,6	5,4	9,8	0	0	0	0	0	0,9	0,8	0	0	0	0
– Golimumab	1,3	2,7	7,8	0	0	0	0	0	0	0	0	1,3	0	1,2
– Infliximab	0,6	1,4	6,8	0	0	0	0	0	0	0,3	0	13	1,2	0
T‑Zell-Kostimulation‑i Abatacept	3,0	0,5	0	0,4	0,3	0,9	0,9	0	0	0	0	0	0	0
BLySi Belimumab	0	0	0	14	0	0,5	0	1,4	0	0	0	0	0	0
IFNαRi Anifrolumab	0	0	0	0,5	0	0	0	0	0	0	0	0	0	0
B‑Zell-Depletion Rituximab	3,4	0,1	0	1,5	1,9	6,3	11	4,8	0	0	29	0,6	0	0
IL-1i: Anakinra	0,2	0	0	0,3	0	0	0,9	0	0,2	0,5	0,3	0	38	15
– Canakinumab	0	0	0	0,1	0	0	0	0	0	0	0	0	13	35
IL-5i: Mepolizumab	0,1^a^	0	0	0	0	0	0	0	0	0	3,9	0	0	0
IL-6Ri	4,6	0,2	0,1	0	2,8	0,5	0	2,8	1,6	39	0,3	0	3,6	0
– Tocilizumab	3,6	0,2	0,1	0	2,8	0,5	0	2,8	1,6	39	0,3	0	1,2	0
– Sarilumab	0,9	0	0	0	0	0	0	0	0	0	0	0	2,4	0
IL-23i und IL-12/23i	0,2	7,2	1,2	0,2	0	0	0	0	0	0	0,3	0	0	0
IL-23i Guselkumab	0,1^a^	4,0	0,5	0	0	0	0	0	0	0	0	0	0	0
Risankizumab	0	0,8	0	0	0	0	0	0	0	0	0	0	0	0
IL-12/23i Ustekinumab	0,1^a^	2,4	0,7	0,2	0	0	0	0	0	0	0,3	0	0	0
IL-17i	0,1	17	9,4	0	0	0	0	0,7	0	0,5	0	0	0	0
– Ixekizumab	0,1^a^	5,5	1,7	0	0	0	0	0	0	0	0	0	0	0
– Secukinumab	0	11	7,3	0	0	0	0	0,7	0	0,5	0	0	0	0
– Bimekizumab	0	0,5	0,3	0	0	0	0	0	0	0	0	0	0	0

Alle Angaben sind Prozentwerte

*RA* rheumatoide Arthritis, *PsA* Psoriasisarthritis, *axSpA* axiale Spondyloarthritis, *SLE* systemischer Lupus erythematodes, *SSc* systemische Sklerose, *SjS* primäres Sjögren-Syndrom, *IIM* idiopathische inflammatorische Myositiden, *MCTD* „mixed connective tissue diseases“, *PMR* Polymyalgia rheumatica, *RZA* Riesenzellarteriitis, *AAV* ANCA-assoziierte Vaskulitiden, *BD* Morbus Behçet, *AOSD* „adult-onset Still’s disease“, *AIE* autoinflammatorische Erkrankungen. *IL* Interleukin, *TNFi* Tumornekrosefaktorinhibitoren, *bDMARD* „biologic DMARD“

^a^Weitere Diagnosen vorliegend

Tocilizumab wurde häufig bei RZA (39 %) eingesetzt, dazu kamen IL-6Ri auch bei 5 % der RA-Patient:innen zum Einsatz. Rituximab war die häufigste bDMARD-Therapie bei AAV (29 %), wurde aber auch bei RA und „off label“ bei Kollagenosen eingesetzt. SLE-Patient:innen erhielten zu 14 % Belimumab und – neu – zu 0,5 % Anifrolumab. Mepolizumab wurde bei 12 von 53 (23 %) der Patient:innen mit einer eosinophilen Granulomatose mit Polyangiitis (EGPA) verordnet.

### tsDMARDs

Januskinaseinhibitoren (JAKi) kamen v. a. bei der RA (11 %) zum Einsatz (s. Tab. 5). Es wurden mehrheitlich Baricitinib (4,1 %) und Upadacitinib (4,4 %), seltener Filgotinib (2,0 %) und Tofacitinib (0,8 %) verordnet.

**Tab. 5:** Zielgerichtete („targeted-synthetic“) DMARDs

	RA	PsA	axSpA	SLE	SSc	SjS	IIM	MCTD	PMR	RZA	AAV	BD	AOSD	AIE
tsDMARD	11	6,4	3,2	0,9	0	0,5	0,9	3,4	0,5	0,5	0,3	7,6	0	0
JAKi	11	4,4	3,1	0,9	0	0,5	0,9	3,4	0,5	0,5	0,3	0,6	0	0
– Baricitinib	4,1	0	0,1	0,3	0	0	0	0,7	0	0	0,3	0	0	0
– Filgotinib	2,0	0	0	0,2	0	0	0	1,4	0	0	0	0	0	0
– Tofacitinib	0,8	0,7	0,4	0,1	0	0	0,9	0	0	0	0	0,6	0	0
– Upadacitinib	4,4	3,6	2,6	0,2	0	0,5	0	1,4	0,5	0,5	0	0	0	0
Apremilast	0	2,0	0,1	0	0	0	0	0	0	0	0	7,0	0	0

Alle Angaben sind Prozentwerte

*RA* rheumatoide Arthritis, *PsA* Psoriasisarthritis, *axSpA* axiale Spondyloarthritis, *SLE* systemischer Lupus erythematodes, *SSc* systemische Sklerose, *SjS* primäres Sjögren-Syndrom, *IIM* idiopathische inflammatorische Myositiden, *MCTD* „mixed connective tissue diseases“, *PMR* Polymyalgia rheumatica, *RZA* Riesenzellarteriitis, *AAV* ANCA-assoziierte Vaskulitiden, *BD* Morbus Behçet, *AOSD* „adult-onset Still’s disease“, *AIE* autoinflammatorische Erkrankungen. *JAKi* Januskinaseinhibitoren, *tsDMARD* „targeted synthetic DMARD“

Apremilast kam bei BD (7 %) und bei PsA (2 %) zum Einsatz.

**Abb. 2:**
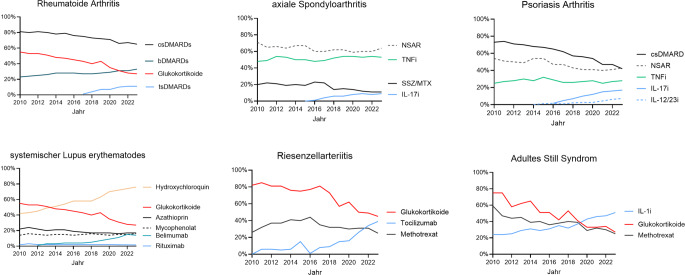
Medikamentöse Therapien in den Jahren 2010 bis 2023. Dargestellt ist der prozentuale Anteil an Patient:innen mit der jeweiligen Therapie. *cs* „conventionel synthetic“, *ts* „targeted synthetic“, *b* „biologic“, DMARD „disease-modifying antirheumatic drugs“, *IL* Interleukin, *NSAR* nichtsteroidale Antirheumatika

Im zeitlichen Verlauf zeigen sich seit 2010 diagnoseübergreifend ein Anstieg an b/tsDMARD-Therapien und ein Rückgang im Einsatz von Glukokortikoiden (s. Abb. 2).

### Ergänzende nichtmedikamentöse Therapiemaßnahmen

In den letzten 12 Monaten erhielten 31 % (RA), 37 % (PsA), 50 % (axSpA) und 20 % (SLE) Physiotherapie. Bei schweren Funktionseinschränkungen gemäß FFbH (< 50) lag der Anteil bei 50 % (RA) bzw. gemäß BASFI (> 4) bei 63 % (axSpA). Ergotherapie erhielten 6 % (SLE) bis 8 % (RA).

### Patientenberichtete Outcomes

Die patientenberichtete Krankheitsaktivität lag krankheitsübergreifend im mittleren Bereich (s. Tab. 6). Der Mittelwert nach der NRS lag bei Werten von 2,7 ± 2,4 (AOSD) bis 4,1 ± 2,4 (SSc). Im Vergleich zur ärztlichen Einschätzung gaben die Betroffenen im Mittel jeweils eine um etwa 2 Einheiten (auf der 0‑ bis 10er-Skala) höhere Krankheitsaktivität an. Schmerzen wurden am stärksten bei axSpA bewertet (4,0 ± 2,3). Der MW von Fatigue war auch bei axSpA, bei SSc, IIM und AIE am höchsten (alle ≥ 4,5). Patient:innen mit IIM und SjS dokumentierten die stärksten Einschränkungen bei Schlafstörungen, psychischem und körperlichem Wohlbefinden.

**Tab. 6:** Patientenberichtete Outcomes nach NRS (0–10), MW (SD)

	RA	PsA	axSpA	SLE	SSc	SjS	IIM	MCTD	PMR	RZA	AAV	BD	AOSD	AIE
Krankheitsaktivität	3,5 (2,4)	3,5 (2,4)	3,7 (2,5)	3,0 (2,4)	4,1 (2,3)	3,9 (2,4)	3,6 (2,5)	3,4 (2,4)	3,5 (2,4)	3,5 (2,7)	2,9 (2,5)	3,1 (2,4)	2,7 (2,4)	2,8 (2,5)
Gesundheitszustand	4,1 (2,2)	4,1 (2,3)	4,1 (2,2)	3,8 (2,3)	4,5 (2,2)	4,3 (2,2)	4,2 (2,1)	3,9 (2,2)	4,2 (2,2)	4,0 (2,2)	3,9 (2,3)	3,9 (2,3)	3,6 (2,2)	3,4 (2,5)
Schmerzen	3,7 (2,5)	3,9 (2,6)	4,0 (2,5)	3,1 (2,7)	3,9 (2,7)	3,7 (2,5)	3,1 (2,7)	3,6 (2,7)	3,4 (2,5)	3,1 (2,6)	2,7 (2,6)	3,0 (2,8)	2,9 (2,4)	2,9 (3,1)
Fatigue	4,2 (2,9)	4,4 (3,0)	4,4 (2,9)	4,3 (3,0)	4,6 (3,0)	4,6 (3,0)	4,7 (2,9)	4,4 (3,0)	3,7 (2,8)	3,8 (2,9)	3,6 (3,0)	3,9 (3,1)	4,0 (3,2)	4,5 (3,1)
Körperliche Schwierigkeiten	3,6 (2,7)	3,7 (2,8)	3,8 (2,7)	3,0 (2,7)	3,9 (2,8)	3,3 (2,7)	3,7 (3,1)	3,4 (2,8)	3,3 (2,6)	3,2 (2,8)	2,8 (2,8)	2,8 (2,8)	2,6 (2,6)	2,8 (3,1)
Schlafstörungen	4,0 (3,1)	4,2 (3,2)	4,1 (3,1)	4,3 (3,3)	4,3 (3,2)	4,4 (3,3)	4,4 (3,3)	4,1 (3,2)	4,0 (3,1)	3,8 (3,1)	3,3 (3,1)	4,1 (3,3)	3,5 (3,1)	3,9 (3,8)
Psychisches Wohlbefinden	3,7 (2,6)	3,7 (2,8)	3,7 (2,7)	3,6 (2,7)	3,9 (2,8)	3,9 (2,7)	4,2 (2,8)	3,4 (2,6)	3,6 (2,6)	3,4 (2,6)	3,1 (2,6)	3,7 (2,9)	3,3 (2,8)	3,8 (3,0)
Körperliches Wohlbefinden	4,1 (2,5)	4,3 (2,5)	4,2 (2,4)	3,8 (2,5)	4,5 (2,5)	4,3 (2,5)	4,4 (2,5)	4,0 (2,6)	3,9 (2,4)	3,8 (2,4)	3,5 (2,5)	3,5 (2,6)	3,9 (2,9)	4,2 (2,6)

Alle Angaben werden auf numerischen Ratingskalen von 0–10 erfasst, 0 ist jeweils der beste Wert und entspricht keiner Einschränkung. Angegeben sind jeweils Mittelwert und Standardabweichung

*RA* rheumatoide Arthritis, *PsA* Psoriasisarthritis, *axSpA* axiale Spondyloarthritis, *SLE* systemischer Lupus erythematodes, *SSc* systemische Sklerose, *SjS* primäres Sjögren-Syndrom, *IIM* idiopathische inflammatorische Myositiden, *MCTD* „mixed connective tissue diseases“, *PMR* Polymyalgia rheumatica, *RZA* Riesenzellarteriitis, *AAV* ANCA-assoziierte Vaskulitiden, *BD* Behçet Disease, *AOSD* „adult-onset Still’s disease“, *AIE* autoinflammatorische Erkrankungen

### Berufliche Teilhabe

Von den Patient:innen im erwerbsfähigen Alter (< 65 Jahre) waren 58 % (SSc) bis 77 % (axSpA) erwerbstätig. Bei allen Diagnosen bis auf IIM waren Männer häufiger erwerbstätig als Frauen, insbesondere der Anteil an Vollzeittätigkeit lag bei Männern deutlich höher. Innerhalb der letzten 12 Monate waren 19 % (PsA) bis 38 % (PMR) arbeitsunfähig mit 14 bis 28 Fehltagen im Median. Eine Erwerbsminderungsrente wegen der rheumatischen Erkrankung hatten 3 % (PMR) bis 18 % (AAV) der unter 65-Jährigen (Tab. 7).

**Tab. 7:** Berufliche Teilhabe bei Patienten unter 65 Jahren

	RA	PsA	axSpA	SLE	SSc	SjS	IIM	MCTD	PMR	RZA	AAV	BD	AOSD
Gesamt, N	2549	983	1018	654	171	122	59	88	96	60	130	111	61
Erwerbstätig (%)	68	71	77	64	58	60	68	63	73	60	64	67	72
– In Vollzeit (%)	45	53	62	37	32	31	39	33	53	32	40	52	43
– In Teilzeit (%)	23	18	15	27	26	30	29	30	20	28	24	15	30
AU im letzten Jahr (%)	20	19	20	20	24	22	32	31	38	^b^	29	21	30
Tage, median	14	18	14	14	17	14	17	14	15	^b^	20	21	28
Berentung Rheuma^a^ (%)	9	7	6	14	16	9	15	17	3	10	18	8	5
Frauen, N	1868	543	386	583	122	113	36	77	48	46	75	51	42
Erwerbstätig (%)	67	66	72	64	56	61	72	65	65	59	58	60	74
– In Vollzeit (%)	38	37	44	34	24	32	33	32	25	22	25	35	33
– In Teilzeit (%)	29	29	29	30	33	31	39	33	40	37	33	25	40
Männer, N	681	440	632	71	49	9	23	11	48	14	55	60	19
Erwerbstätig (%)	71	78	80	65	64	^b^	61	^b^	81	^b^	73	73	^b^
– In Vollzeit (%)	65	73	74	58	54	^b^	48	^b^	81	^b^	62	67	^b^
– In Teilzeit (%)	6	5	7	7	10	^b^	13	^b^	0	^b^	11	7	^b^

^a^Berentung aufgrund der rheumatischen Erkrankung

^b^Bei kleinen Fallzahlen keine Angabe

## Diskussion

Die Kerndokumentation liefert seit 30 Jahren aktuelle Daten zur Versorgung von Patient:innen mit entzündlich rheumatischen Erkrankungen, die sich in fachärztlicher rheumatologischer ambulanter Behandlung befinden.

Hinsichtlich der medikamentösen Therapie zeigt sich die kontinuierliche Erweiterung des rheumatologischen Therapiespektrums v. a. im Bereich der Interleukininhibitoren: IL-1i und IL-1Ri werden bei AOSD und AIE, IL-5i bei EGPA, IL-6Ri bei RZA und RA, IL-12/23i und IL-23i bei PsA und IL-17i bei PsA und axSpA eingesetzt. Dazu kommen neben den TNFi noch die Inhibition von BLyS, die TypI-IFNR-Blockade sowie Kostimulationsblockade und B‑Zell-Depletion. Der Einsatz von GC ist seit 2010 diagnoseübergreifend deutlich rückläufig, während spezifische Therapien mit Biologika und tsDMARDs zunehmend eingesetzt werden. Weitere Trends können über die Versorgungstrends der Kerndokumentation verfolgt werden [3].

Bei den patientenberichteten Outcomes gibt es diagnosespezifische Unterschiede im Schweregrad der Angaben. Insgesamt zeichnet sich im Vergleich mit früheren Jahren kaum eine Verbesserung der Parameter ab, selbst bei den Diagnosen wie RA und axSpA, die heute gut medikamentös behandelt werden können. Bei der Interpretation muss aber berücksichtigt werden, dass die Kerndokumentation ein Kollektiv langjährig erkrankter Patient:innen abbildet (die mediane Krankheitsdauer liegt für die meisten Diagnosen bei über 10 Jahren) und derzeit nur wenige Neuerkrankte dokumentiert werden. Ob eine frühe intensive Therapie auch die PROs nachhaltig verbessern kann, lässt sich anhand dieser Daten nicht beantworten.

Die berufliche Teilhabe bei Patient:innen mit Vaskulitiden und Kollagenosen ist mit insgesamt etwa 64 % noch geringer als bei den Arthritiden mit 68–77 %, was die noch nicht ausreichende Verfügbarkeit von effektiven biologischen bzw. zielgerichteten Therapien bei diesen Diagnosen widerspiegelt. Eine ausführliche Analyse der Erwerbstätigkeit im Vergleich zu Bevölkerungsdaten wurde kürzlich publiziert [4].

### Limitationen und Stärken

Die Kerndokumentation umfasst mit knapp 14.000 Patient:innen etwas weniger als 1 % der geschätzten Zahl an Menschen mit einer entzündlich rheumatischen Erkrankung in Deutschland. Alle Patient:innen aus der Kerndokumentation kommen aus Rheumazentren mit hoher Expertise und bilden daher eine fachärztliche Versorgung auf sehr hohem Niveau ab. Durch die bundesweite Teilnahme von Einzelpraxen, MVZ, Krankenhäusern und Universitätsambulanzen bildet die Kerndokumentation unterschiedliche Versorgungssektoren und verschiedene Regionen mit Großstädten bis zu ländlichen Regionen ab. Die Versorgung von Betroffenen, die nicht rheumatologisch betreut werden, kann jedoch nicht abgebildet werden. Aus Abrechnungsdaten der gesetzlichen Krankenversicherung ist bekannt, dass ohne Rheumatologenkontakt z. B. die Verordnungshäufigkeit von DMARDs deutlich niedriger ist [5].

## Fazit

Der Jahresbericht 2025 aus der Kerndokumentation zeigt die Vielfältigkeit der rheumatischen Diagnosen und das kontinuierlich wachsende Therapiespektrum, welches wir einsetzen können. Die gleichzeitige Erfassung ärztlicher und patientenberichteter Daten gibt einen umfassenden aktuellen Einblick in die rheumatologische Versorgungsqualität unserer Patient:innen.
